# MicroRNA as New Tools for Prostate Cancer Risk Assessment and Therapeutic Intervention: Results from Clinical Data Set and Patients' Samples

**DOI:** 10.1155/2014/146170

**Published:** 2014-09-16

**Authors:** Alessio Cannistraci, Anna Laura Di Pace, Ruggero De Maria, Désirée Bonci

**Affiliations:** ^1^Departement of Hematology, Oncology and Molecular Medicine, Istituto Superiore Sanità, Viale Regina Elena 299, 00161 Rome, Italy; ^2^Regina Elena National Cancer Institute, Via Fermo Ognibene, 00144 Rome, Italy

## Abstract

Prostate cancer (PCa) is one of the leading causes of cancer-related death in men. Despite considerable advances in prostate cancer early detection and clinical management, validation of new biomarkers able to predict the natural history of tumor progression is still necessary in order to reduce overtreatment and to guide therapeutic decisions. MicroRNAs are endogenous noncoding RNAs which offer a fast fine-tuning and energy-saving mechanism for posttranscriptional control of protein expression. Growing evidence indicate that these RNAs are able to regulate basic cell functions and their aberrant expression has been significantly correlated with cancer development. Therefore, detection of microRNAs in tumor tissues and body fluids represents a new tool for early diagnosis and patient prognosis prediction. In this review, we summarize current knowledge about microRNA deregulation in prostate cancer mainly focusing on the different clinical aspects of the disease. We also highlight the potential roles of microRNAs in PCa management, while also discussing several current challenges and needed future research.

## 1. Introduction

**Figure 1:**
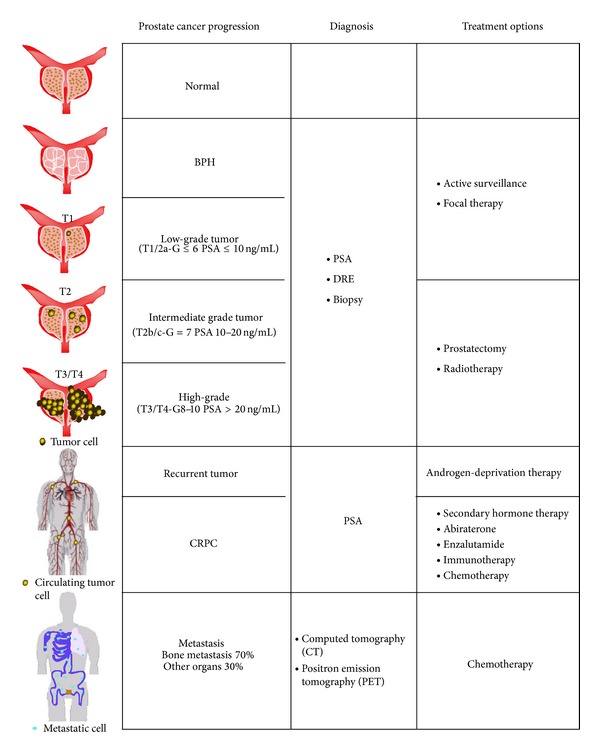
Representative scheme of prostate cancer tumor progression.

In developed Western countries, prostate cancer is the most common solid tumor diagnosed in men and one of the highest causes of cancer-related deaths after lung cancer [1]. For many years, digital rectal examination (DRE) represented the primary diagnostic test for prostate cancer detection. In the late 1980s, prostate specific antigen- (PSA-) screening was rapidly and widely adopted for PCa diagnosis [2]. However, in spite of the significant improvement in early detection and relapse assessment after radical prostatectomy, there is no evidence that the PSA-test reduces the risk of death for the disease. In fact, serum PSA level may be a consequence of different variable events, such as benign prostatic hyperplasia (BPH), inflammation of the gland, or pharmacological therapy, and it is not correlated with either predicting tumor aggressiveness or therapy responsiveness. Thus, PSA level evaluation inevitably affects the false-positive rate of prostate cancer detection, leading to overdiagnosis of patients who present nonneoplastic alteration of the prostate gland or clinically insignificant cancer [3, 4]. As a consequence of its low predictive value, PSA screening has caused extra diagnosis and overtreatment in cancer patients who are subject to invasive or radical procedures with significant side-effects and without effective benefits in quality of life. In the last few years, several active surveillance protocols have been approved for monitoring patients with low risk cancers [5]. This approach may strongly reduce patients' overtreatment and morbidity associated with surgery. However, a considerable group of low-risk patients may experience tumor progression. In this case, radical prostatectomy and radiotherapy represent the standard treatment for localized high-grade tumors [6, 7]. Nevertheless, a significant percentage of radical-treated patients (30–35%) may develop biochemical recurrence, with rising levels of PSA as a consequence of the presence of cancer cells [8]. Since PCa depends on hormone signaling for its growth and survival, androgen-deprivation therapy represents the first-line therapy for this stage of the disease, with significant improvement in patient prognosis. However, within 2 years of treatment a significant percentage of these patients develop a castration-resistant form (CRPC) of the disease, which is ultimately responsible for PCa mortality [9]. Bone metastases occur in 70% of castration-resistant patients and are associated with impairment in quality of life due to the onset of skeletal-related events (SREs) such as pathologic fractures, spinal cord compression, need of surgery or radiotherapy on bone, hypercalcaemia, and bone pain (Figure 1).

Therefore, the identification of predictive biomarkers able to discriminate indolent tumors from aggressive ones would be helpful in reducing the risk of overdiagnosis, avoiding patients undergoing surgical/radiation therapies without any clear clinical benefits but complex side effects. Moreover, the consequent possibility to stratify patients on the basis of their responsiveness to treatment would be helpful in guiding therapeutic decisions and in paving the way to personalized medicine.

MicroRNAs (or miRs) are a family of small noncoding RNA which are able to regulate gene expression at different levels [10]. miRs are interspersed in the genome as independent transcriptional units or within the open reading frame of a specific gene. They are transcribed by the RNA polymerase II and are processed through a series of endonucleolytic cleavages, from nucleus to cytoplasm, in a mature form of 22–25 nucleotide fragments which are able to regulate mRNA spatial and temporal translation/degradation through association with the RNA-induced silencing complex (RISC complex). Generally, sequences recognized by miRs are located in the 3′-untranslated region (3′-UTR) of coding RNA but several studies demonstrated that microRNAs can also bind to the 5′-UTR [11] and to the coding sequence [12] maintaining their regulatory properties. Furthermore, it has been shown that these small RNAs play a dual role in cellular regulation not only in inhibiting but also in activating gene expression through direct binding to target RNA [13]. Based on these observations, which highlight the complexity of this fine-tune control of protein expression, it is not surprising that microRNAs are involved in all basic cell processes, such as proliferation, stemness maintenance, differentiation, and apoptosis. Consequently, deregulation of their expression is significantly correlated with the etiology of many diseases, including cancer. The first evidence of microRNA's role in cancer came from the study of Calin and colleagues in 2002 [14]. They demonstrated that miR-15a and miR-16a were either absent or downregulated in approximately 70% of patients with B-cell chronic lymphocytic leukemia (B-CLL), due to the deletion of chromosome region 13q14.

Circulating microRNAs represent the new expanding field in the world of biomarker research. Indeed, they potentially epitomize the perfect candidates for diagnostic and prognostic purpose [15–17], since their expression reflects the molecular profile of tumor origin [18], and they are highly stable in body fluids and resistant to storage handling. In fact, it has been demonstrated that serum miRs remain stable after being subjected to severe conditions (boiling, high/low pH levels, and freeze-thaw cycles) [19] and preserved in long-term banked human serum samples. Although the mechanisms by which circulating microRNAs are protected from RNase digestion have not yet been extensively described, an increasing amount of evidence indicates that miRs can form complexes with RNA-binding proteins [20] or can be incorporated in cell-derived secreted exosomes [21]. In the tumor environment, tumor-derived exosomes act as carriers of genetic information and proteins destined to recipient cells, where their production is finely regulated by cancer cells. Thus, the incorporation of specific microRNAs in these microvescicles would not be exclusively considered a stochastic process due to the saturated levels of intracellular RNA, but also a functional molecular program which permits cancer cells to influence homeostasis of the surrounding microenvironment. A recent study gave further evidence of this mechanism, demonstrating that exosomes-mediated transfer of miR-105 efficiently destroys tight junctions of the vascular endothelial monolayer, prompting tumor metastasis [22]. Currently, a variety of circulating RNA detection methods is available but the most commonly used are Real-Time PCR, microarray, and deep sequencing [23]. Although, several technical biases are associated with each of these detection protocols including contamination of microRNA samples from other cellular sources (blood cells and stroma cells), there is a lack of unequivocal endogenous control due to the cell-free conditions, specificity of probes and enzyme efficiency rate. Consequently, published studies report conflicting data indicating the necessity of a standardized and robust method with universal parameters for circulating microRNA analysis. From this point on, our knowledge on microRNA-cancer connection has increased exponentially but it is far from complete. However, it is evident that microRNA analysis represents an innovative and specific tool for the improvement of diagnostic, prognostic, and therapeutic protocols. As a consequence of this assumption, an increasing number of studies analyzing genomic alteration of large cohorts of patients are evaluating also the microRNA expression profiles, producing a high quantity of data which could be useful to improve cancer management [24, 25]. Our review focuses on miR clinical relevance and how much our knowledge is expanding in this field. We selected all articles reporting applications and results obtained analyzing patient-derived samples, with a minor analysis of papers clearly oriented towards basic research.

In order to provide a better understanding of recent advances in miR-based biomarker validation for PCa, we divided articles into three main topics: (1) diagnosis, (2) prognosis, and (3) therapy.

## 2. MicroRNAs and PCa Diagnosis

In 2014, an estimate of 233,000 new cases of PCa will be diagnosed with a 12% mortality rate (National Cancer Institute, http://www.cancer.gov/). A significant percentage of patients who experience tumor development before the mean age represent the most difficult group to manage from a clinical and psychological perspective. Several approaches have been proposed to improve cancer diagnostic accuracy of the PSA test, including the measurement of PSA velocity (change over time), PSA density (ratio between protein blood-level and prostate volume), and PSA-free and protein-bound PSA levels. However, the clinical usefulness of these strategies remains solely experimental. Sensitive biomarkers are needed in order to reduce overdiagnosis, overtreatment, biopsy side effects, and psychological stress [26]. Since microRNA expression reflects the tumor of origin [27] and that it has been correlated with prostate cancer development and progression [28–30], miRs represent an intriguing and promising approach for improving specificity of diagnosis. To date, a distinctive microRNA signature able to distinguish between healthy and diseased patients has not been found but encouraging results have been obtained for PCa (Table 1). In 2006, Volinia and colleagues analyzed the expression profile of 228 miRs in 56 prostate tumor tissues and 7 normal controls [31]. Upregulation of miR-32, -26a, -181a, -93, -196a, -25, -92, and let-7i was confirmed also by Ambs [32] in a cohort of 76 microdissected tissues including 60 tumor specimens and 16 controls. In this study, the authors identified miR-101, -30c, and -195 significantly upregulated in patients with extraprostatic extension of cancer cells, suggesting a possible role in predicting the evolution of the disease. Interestingly, subsequent studies demonstrated the tumorigenic role of miR-181a and miR-196 in several types of cancers, regulating fundamental processes of malignant progression, such as epithelial to mesenchymal transition (EMT) [33–35] and invasive properties of the cells [36, 37].

In contrast to the previously described studies, Porkka and colleagues demonstrated a significant downregulation of microRNA expression levels correlating with PCa progression [38]. They evaluated a panel of 13 clinical prostate tissues, including 4 BPH, 5 hormone-naïve, and 4 hormone-refractory PCa tumors. Their analysis revealed 37 downregulated and 14 upregulated miRs in PCa specimens. Among downregulated microRNAs miR-16, miR-99, and let-7 family are well known tumor-suppressor genes [39–42]. Interestingly, reduced levels of miR-205, miR-100, and miR-30 family were observed only in hormone-refractory specimens suggesting a hypothetical prognostic role for CRPC prediction. Porkka's profile discretely overlapped the one generated by Ozen et al. [43]. In the latter study, the authors observed a significant reduction in miR levels in 16 prostate cancer tissues compared with 10 normal prostate tissues. Among the 85 detectable miRs, 76 were downregulated, with a tendency toward a more global downregulation of miRs in case of early PSA recurrence. Stroma contamination could be one possible explanation for widespread downregulation of miRs in prostate cancer tissues. In fact, based on Ozen hypothesis, normal stromal tissues express higher levels of miR and thus, since cancers have less stroma than normal tissues, the relative expression of miRs would appear to be decreased. In line with this hypothesis, a more recent study confirmed the general downregulation of microRNA expression correlating with tumor progression [44]. The authors analyzed a group of 102 patient-derived tissues through microarray. Deregulation of 54 microRNAs was found to clearly segregate PCa specimens from normal adjacent tissues. Moreover, a panel of 25 miRs (13 downregulated and 12 upregulated) significantly correlated with poor clinical parameters, such as Gleason score, incidence of metastases, and E-Twenty-Six variant 1 (ETV1) alterations. Among the microRNAs deregulated in the aforementioned study, 13 of them were specifically analyzed by Larne and colleagues in a cohort of FFPE prostatic tissues derived from 49 prostate cancer patients and 25 men without PCa [45]. Single-assay qRT-PCR analysis revealed a signature of 7 deregulated microRNAs (miR-96-5p, -183-5p, -183-3p, -145-5p, -205-5p, -221-5p, and -409-5p) differentially expressed in PCa samples compared with healthy control. The most significant upregulated (miR-96-5p and miR-183-5p) and downregulated (miR-145-5p and miR-221-5p) microRNAs were combined together in order to obtain an miR index quote (miQ) which was able to discriminate with high accuracy prostate cancer from nonprostate cancer samples and to significantly predict tumor aggressiveness and patients' metastatic status. The predictive value of miQ was further validated in four different cohorts and, despite the differences in size, methodology, and experimental design, the results obtained indicate that miQ could represent a useful clinical biomarker reducing the intervariability between samples.

In recent years, the discovery of circulating microRNAs has resulted in a welcome and exciting change in biomarker research. Particularly for prostate cancer, the possibility to substitute invasive procedures, such as DRE and biopsy, will certainly improve patient care. In 2011, Moltzahn et al. compared microRNA serum levels of 12 healthy men and 36 PCa patients, divided in low risk, intermediate risk and high risk based on the CAPRA score [46, 47]. Ten miRs were substantially different between the healthy and all malignant samples. Four were downregulated in the cancer group (miR-223, -26b, -30c, and -24), and 6 were upregulated in the cancer group (miR-20b, -874, -1274a, -1207-5p, -93, and -106a). Two miRs shown a linear correlation between miR levels and cancer risk: miR-24 steadily decreased with risk, whereas miR-106a steadily increased with risk. A similar analysis was conducted by Bryant and colleagues in 2012 [48]. Using a qRT-PCR microarray panel, they analyzed changing in microRNA expression profiles comparing 78 plasma samples derived from prostate cancer patients and 28 healthy controls. A signature of 12 microRNAs was found to be significantly deregulated in PCa specimens and the upregulation of miR-107 and miR-574-3p in localized prostate cancer patients was also validated through single-assay analysis. Notably, these two microRNAs were present at significantly higher concentrations in the urine of men with cancer compared with the control, indicating their potential as noninvasive detectable biomarkers. Because of the ease of collection, and the fact that prostate cells are directly released into the urethra through prostatic ducts after DRE, urine has become the future for noninvasive biomarker testing. From the first study of Bryant, much effort has been devoted to microRNA detection in patients' urine seeing a rapid expansion in this branch of research. In 2013, Srivastava and colleagues analyzed the expressions of miR-205, miR-214, miR-221, and miR-99b in 36 PCa patients and 12 age and ethnicity matched healthy men [49]. miR-205 and miR-214 were found to be significantly downregulated in cancer samples compared with normal controls. More recently, a cohort of 30 patient-derived urine samples (8 PCa patients, 12 BPH patients, and 10 healthy men) were analyzed for their microRNA expression profiles [50]. From the deregulated group, a panel of 7 miRs (miR-1234, -1238, -1913, -486-5p, -1825, -484, and -483-5p) was selected for further analysis. Single-assay evaluation showed a significant modulation of miR-1825 and miR-484 which were, respectively, upregulated and downregulated in PCa samples compared with healthy controls. In the BPH group, the same trend was observed only for miR-484 whereas upregulation of miR-1825 was found to be variable among the samples. The expression pattern observed for these two miRs was not confirmed when patients were reevaluated two years later but combining data from microRNAs deregulation and abnormal PSA levels, the presence of prostate cancer was detected with 40% of sensitivity and 81% of specificity. These promising results testify that biomarkers detectable in body fluids, which can be obtained in a noninvasive manner, seem a good alternative as possible screening tool.

## 3. MicroRNAs and PCa Prognosis

Patient prognosis prediction and follow-up monitoring still represent the major challenges for clinical management of prostate cancer. Prostate specific antigen-screening has significantly improved tumor early detection and relapse assessment after radical prostatectomy. However, serum PSA level is not directly correlated with tumor aggressiveness or therapy sensitiveness and its usefulness in reducing cancer mortality rate is still under debate. MicroRNAs are gaining considerable attention in the clinical setting because their expression seems to accurately reflect the malignant evolution of cancer cells [27]. Moreover, the high stability in frozen and formalin-fixed and paraffin-embedded (FFPE) tissues [51] combined with the possibility to detect these small RNAs in body fluids, including serum, plasma, urine, and saliva [21], make them highly attractive as potential biomarkers. Based on this evidence, the number of studies focusing on microRNA expression profiles in prostate cancer has notably increased; however, results deriving from high-throughput approaches produced partially contradictory reports. Lack of uniformity in proposed datasets for patient stratification is in part due to the different study design, underestimated treatments of the patients, methods of sample collection, presence of contaminating cells, and sensitivity and specificity of the platforms used. A summary of the most prognostic significant miR has been reported in Table 2. As a consequence of an abundant data in literature and extreme complexity of the discussion in prognostic values of the data, we divided this paragraph into two sections, one dedicated to tissue and the other to fluid samples.

### 3.1. Prognostic MicroRNAs in PCa Tissues

The first attempt to segregate patients through a microRNA profile screening came from primary tumor analyses. Although it may be questionable whether tumor tissue evaluation effectively predicts changes observed in cancer cells, which are not only spatial but also temporally regulated, the availability of a limitless quantity of banked tumor samples makes them a unique resource of information. The possibility of analyzing preserved specimens for retrospective studies is particularly advantageous for PCa, which is a slow-growing disease requiring a long-scheduled follow-up monitoring program to obtain significant correlation between biomarker expression and tumor progression.

#### 3.1.1. Biochemical Recurrence (BCR) and MicroRNAs

Biochemical recurrence is defined as* de novo* rising of PSA blood levels after radical prostatectomy [52]. BCR is widely used as an early end point to assess treatment success and frequently prompts the initiation of secondary therapy in order to reduce the risk of metastasis formation. Several studies were attempted in order to establish a distinctive signature of microRNAs able to stratify patients on the basis of their risk in developing BCR. However, much controversy is still present in the literature possibly due to several biases (study design, sample collections, sensitivity, and specificity of platforms used) which are common in translational research. In 2009, Tong and colleagues analyzed the microRNA expression profiles of 40 FFPE tumor tissues divided in early biochemical relapse (*n* = 20) and nonrelapsed (*n* = 20) patients [53]. A signature of 16 microRNAs was able to segregate 75% of analyzed relapsed patients, excluding 85% of patients with no evidence of recurrence. Interestingly, single-assay qRT-PCR validates the upregulation of miR-16, miR-135b, miR-194, and miR-218 and downregulation of miR-140. In addition, the authors showed a significant reduction of miR-23b, -100, -145, -221, and -222 in prostate cancer tissues compared with normal adjacent tissues, giving a diagnostic relevance to their analysis. Another study performed by Schaefer and colleagues [54], identified a microRNA signature of 10 miRs downregulated (miR-16, -31, -125b, -145, -149, -181b, -184, -205, -221, and -222) and 5 upregulated (miR-96, -182, -182*, -183, and -375), differently expressed in tumor tissues compared with normal adjacent tissues in a cohort of 76 prostate cancer patients. Further validation experiments significantly correlate miR-31, -125b, -205, -222, and -96 with Gleason score and tumor stage. miR-96 expression was also associated with BCR, indicating its potential role as a prognostic biomarker. Notably, negative modulation of miR-205 in recurrent samples was confirmed also by Hulf et al. [55] who also demonstrated that this microRNA can impair cell viability of cancer cells through modulation of MED1 which has been correlated with castration-resistance acquisition [56]. Due to the high number of studies demonstrating the downregulation of miR-205 in patient-derived samples, the role of miR-205 in PCa biology has been further investigated in basic research, and recent reports demonstrated that this microRNA exerts its tumor-suppressive functions directly inhibiting the expression of the AR and its downstream signaling cascade, c-SRC oncogene and the antiapoptotic Bcl-2 protein [57–60]. Interestingly, Mittal and colleagues observed that the delivery of gemcitabine-conjugated and miR-205—complexed copolymers effectively reverses chemoresistance, invasion, and migration of pancreatic cancer cells and inhibits tumor growth using* in vivo* model [61]. All these results demonstrate the clinical relevance of microRNAs as therapeutic tools for treating cancer diseases and the significant advances in nanotechnology applied to medicine with a consequent increase in the number of curative options for diseased patients. Among downregulated microRNAs correlating with PCa cancer progression, let-7b and let-7c were demonstrated to be lost in patients with shorter disease-free survival time [62]. This result is in agreement with the demonstrated biological role of let-7c in suppressing the expression of androgen receptor (AR) in prostate cancer cells [63]. Interestingly, the let-7c/AR interaction was not mediated by the canonical mechanism of miR-mediated control of gene expression, but rather through the targeting of v-myc avian myelocytomatosis viral oncogene homolog (MYC), a recognized transcription factor for the AR. The microRNA—mediated regulation of the androgen receptor represents a new attractive way trying to unravel the different pathways, in which this molecule plays a pivotal role, and which are responsible for PCa progression. However, since we focused mainly in the clinical aspects of microRNAs biology, we decided not to discuss papers lacking in analyzing clinical samples. Nevertheless, in order to facilitate the comprehension of the importance of AR targeting for future perspectives, we listed in Table 3 several microRNAs involved in receptor regulation that have been validated in basic research studies.

Several microRNAs play a dual role in prostate cancer development and sometimes show opposite behavior in basic research compared to translational studies. This is the case of miR-100 and miR-221/-222. Basic research studies have demonstrated the tumor-suppressor function for miR-100, regulating several oncogenes, such as insulin-like growth factor 2 (IGF2) and mechanistic target of rapamycin (mTOR) [64, 65]. However, Leite et al. in 2011 [66] found a significant overexpression of these microRNAs in BCR prostate cancer samples. The same scenario is applicable to miR-221/-222 which are well known oncomiRs acting in various tumor types [67] and regulating cyclin-dependent kinase (CDK) inhibitors p27^Kip1^ and p57^Kip2^ [68, 69]. Moreover, the work of Sun et al. [70] indicated that miR-221/-222 are able to reduce the sensitivity of androgen-dependent cell lines to dihydrotestosterone (DHT) resulting in androgen independence acquisition. All these results contradict other various studies reporting a significant downregulation of miR-221/-222 expression in clinical samples representative of PCa progression, including castration-resistant and biochemical recurrence stages [38, 54, 71]. More recently, Karatas and colleagues described a significant downregulation of miR-1 and miR-133b in recurrent compared with disease-free patients [72]. As negative modulation of miR-1 is in agreement with its tumor-suppressive role in prostate cancer cells, modulating at least indirectly the expression of AR and consequently their proliferative capabilities [73], downregulation of miR-133b does not reflect recent reports in which its dependence on androgen receptor and its role in maintaining cell viability is described [74, 75].

Altogether, these observations demonstrated the fundamental role of the biological context in which microRNAs are located but, at the same time, they stress the need to bridge the gap between basic and translational research in order to optimize the efforts toward improving cancer management.

#### 3.1.2. Castration Resistant Prostate Cancer (CRPC) and MicroRNAs

The concept of androgen deprivation for the treatment of advanced prostate cancer was developed by Huggins and Hodges in 1941 [76], and up until today, AR—target therapy remains the first line treatment for this disease. Almost all patients with advanced prostate cancer initially respond to androgen deprivation therapy (ADT), showing reduced PSA levels indicating a partial regression of residual tumor. However, this type of condition is transitory and invariably develops into a castration-resistant form which leads to the formation of bone metastases in a significant percentage of treated patients.

miR-21 is one of the most commonly deregulated oncomiR in cancer [77]. Validated targets of miR-21 enclose several genes mainly implicated in suppressing cell migration and invasion, including programmed cell death 4 (PDCD4), phosphatase and tensin homolog (PTEN), tropomyosin 1 (TPM1), sprouty homolog 2 (SPRY2) and the metalloprotease inhibitors TIMP3 and reversion-inducing-cysteine-rich protein with kazal motifs (RECK) [78–83]. In PCa biology, miR-21 expression increases together with clinical parameters (pathological stage, lymph node metastasis, capsular invasion, organ confined disease and Gleason score) and it is correlated with biochemical relapse, castration resistance and metastasis formation [66, 84, 85]. Significantly, an increase of miR-21 and miR-145 in D'Amico et al. score [86] high-risk compared with low-risk patients, were described by Shen et al. [87]. miR-21 was also described as predictive biomarker of PCa progression since its blood-levels are directly correlated with castration-resistant and metastatic states [88]. One of the molecular mechanisms through which miR-21 is able to regulate castration resistance process was described recently. It involves the activation of the epithelial to mesenchymal transition (EMT), through negative modulation of tumor-suppressor BTG2 [89]. A further microarray analysis identified a panel of miRs (miR-21, -32, -99a, -99b, -148a, -221, and -590-5p) differentially expressed in castration-resistant tumors compared to benign prostate hyperplasia [90], and the functional study revealed that also miR-32 can inhibit BTG2 expression, suggesting a prognostic and therapeutic role of this miR.

EMT seems to be a relevant process in escaping blockade of androgen signals. miR-205 and miR-30 are two tumor-suppressor microRNAs which can block the transition of cancer cells towards an undifferentiated and more aggressive state and both miRs have been negatively correlated with prostate cancer malignant evolution. miR-30 reduces expression levels of v-ets avian erythroblastosis virus E26 oncogene homolog (ERG) gene, which is one of the EMT-associated effectors and, more importantly, is the most frequently overexpressed oncogene in PCa activated by genomic fusion of TMPRSS2 and ERG genomic loci [91]. In the same way, miR-205 exerts its functions inhibiting the translation of EMT-related genes zinc-finger-E-box-binding homeobox 1 (ZEB1) and 2 (ZEB2/SIP1) [92]. Moreover, its ectopic expression in prostate cancer cell can impair dedifferentiation and invasive properties acquisition blocking cancer associate fibroblast (CAF) stimulation [93].

#### 3.1.3. Bone Metastasis and MicroRNAs

Bone metastasis is a common and severe complication of late-stage prostate cancer. Complex interactions between tumor cells, bone cells, and a milieu of components in their microenvironment contribute to the osteolytic, osteoblastic, or mixed lesions present in patients with advanced forms of PCa. Despite the enormous efforts in unraveling the molecular mechanisms regulating bone metastasis, this remains the most clinically relevant but poorly understood aspect of the disease.

Analyzing a cohort of 13 patient-derived specimens (6 primary tumors and 7 bone metastatic PCa samples), Peng and colleagues identified 5 microRNAs (miR-508-5p, -143, -145, -33a, and -100) significantly decreased in bone metastasis [94]. miR-143 and miR-145 were also found to be inversely correlated with serum PSA levels and Gleason score. Further analysis in metastatic PC3 cell line demonstrated that overexpression of these two miRs can reduce invasive capabilities in increasing expression of E-Cadherin and consequently impairing EMT activation.

Similar results were obtained for miR-203 whose expression was significantly attenuated in bone metastatic tissues compared with normal tissues [95]. As a possible explanation for this modulation, the authors demonstrated that miR-203 controls the expression of the EMT factor ZEB2 and the bone metastasis-related factor RUNX2. Moreover, this miR has been associated with “stemness” maintenance owing to its ability to inhibit the self-renewal-associated BMI1 polycomb ring finger oncogene (BMI1) [96].

Although the significant association between miR-15/-16 cluster and bone metastasis formation has not yet been confirmed, the role of these two microRNAs in PCa, and other cancer types, has been extensively evaluated [39]. Alteration of the miR-15a and miR-16-1 translates into multiple tumor-promoting processes through the derepression of key cell cycle-and apoptosis-related genes such as B-cell CLL/lymphoma 2 (BCL2), wingless-type MMTV integration site family-member 3A (WNT3A) and cyclin D1 (CCND1) [97]. miR-15 and miR-16 have been found to be downregulated also in cancer-associated fibroblasts (CAFs), promoting malignant transformation and progression [98]. Reduced miR-15a and -16-1 levels in CAFs result in FGF-2/FGFR1 axis activation, which ultimately increases the tumor-supportive capabilities of CAFs. Interestingly, delivery of synthetic miR-16 was shown to be able to inhibit the growth of metastatic prostate cancer cell lines in mouse bone [99]. This study not only suggests that loss of miR-16 is a predictor factor of metastatic colonization of bones but also that systemic delivery of miR-16 could represent a novel type of personalized-therapy for treating patients with advanced prostate cancer.

### 3.2. Prognostic MicroRNAs in Patient Body Fluids Circulatory System

The first report regarding the potential role of circulating microRNAs as predictive biomarkers in PCa, was published by Mitchell and colleagues in 2008 [19]. They analyzed a panel of six candidates miRs (miR-100, -125b, -141, -143, -296, and -205) in serum samples collected from 25 metastatic prostate cancer patients and 25 age-matched healthy donors. Among all the candidates, only miR-141 showed a significant overexpression in PCa patients and a further analysis revealed a moderate correlation with PSA levels. Similar results were obtained by Brase et al., who demonstrated that miR-141 is upregulated, together with miR-375, in sera of metastatic patients compared with nonmetastatic samples [100]. Interestingly, a significant deregulation of these two miRs was found within the nonmetastatic group (low-risk versus high-risk), indicating their potential role not only in prognosis but also in early diagnosis. The upregulation of miR-141 and miR-375 in plasma and serum samples of metastatic patients was also demonstrated in the aforementioned study of Bryant et al. [48]. The biological role of miR-141 in metastatic progression is still not fully understood, however its expression has been demonstrated to be positively correlated with expression of alkaline phosphatase (ALP), a marker of skeletal lesions and with increased level of bone metastatic lesions [101].

Circulating microRNAs can also be associated with biochemical recurrence and castration-resistant state acquisition. In 2013, Selth and colleagues revealed that miR-194 and miR-146-3p are overexpressed in sera of patients experiencing biochemical relapse [102]. Interestingly, they found also an increased level of miR-141, miR-375, and miR-200 in these patients but further validation analyses did not confirm a significant statistical relevance. On the contrary, in the study conducted by Nguyen et al., miR-141 and miR-375 were found strongly upregulated in a cohort of patients experiencing CRPC compared with localized PCa patients [103]. The same authors observed a significant correlation with castration resistant state also for upregulated miR-378* and for downregulated miR-409-3p. All these results, especially those associated with miR-141 and miR-375, confirmed that microRNA expression is extremely sensitive to molecular changes in cancer cells.

Similarly, miR-21 has been reported to be upregulated in CRPC patients. Zhang and colleagues analyzed a cohort of 6 BPH patients, 20 patients with localized PCa, 20 androgen-deprivation therapy responsive patients and 10 CRPC patients under docetaxel treatment, observing a positive correlation between miR-21 levels and tumor progression [88]. Intriguingly, further observations in the CRPC cohort revealed a direct correlation between high expression of miR-21 and unresponsiveness to chemotherapy, indicating that this microRNA can distinguish between patients that will benefit from the chemotherapeutic regimen and patients that will not.

## 4. MicroRNAs and PCa Therapy

In addition to the absence of reliable diagnostic and prognostic indicators, prostate cancer management is also impaired by the lack of tools in guiding treatment assignment and evaluating therapy response. Testosterone suppression represents the gold-standard first line treatment for men with recurrent PCa, but the majority of tumors evolve towards a castration-resistant state (CRPC) within 2 years. Despite such tangible advances in local and systemic therapy, the global management of PCa patients is still far from ideal, and many questions concerning the optimal treatment of both early and advanced forms still need to be addressed. The administration of targeted or conventional therapies requires accuracy of staging procedures and predictive biomarkers of prostate cancer patients response. Hormone therapy for prostate cancer is typically initiated using drugs that lower serum testosterone, often in combination with competitive androgen receptor antagonists, such as bicalutamide or Casodex (CDX). The initial response to ADT is significantly high in almost all treated patients but, within two years after the initial treatment, a significant group of these patients fails to be sensitive to this kind of treatment. Once this occurs, secondary hormone therapy is usually considered and it includes different types of antiandrogens. Recently, different drugs able to block androgen synthesis have been approved by the FDA, such as (i) Abiraterone Acetate (AA) which is a potent and selective inhibitor of CYP17, a protein required for androgen biosynthesis in the testes, adrenal glands and prostate tissue, and (ii) enzalutamide (Xtandi) which blocks the effects of androgens in stimulating the growth of the prostate cancer cells. Although initially effective at blocking tumor growth, these therapies eventually fail, leading to a lethal drug-resistant condition, castration-resistant state. For men unresponsive to all forms of hormone treatment, the clinical protocol actually provides different therapeutic approaches, such as bisphosphonates administration, targeted therapies, immunotherapy (Sipuleucel-T), and chemotherapy. Recently, Radium-223 (Xofigo) has been approved as a new agent for bone metastasis treatment. It is a radioactive element that localizes in bone and its delivery may be effective at relieving bone pain, preventing complications and prolonging life expectancy [104]. The availability of biomarkers that discriminate patients with indolent or aggressive tumors would allow appropriate treatment early tumors doomed to become aggressive and metastatic. New molecular markers of therapy response will be essential in driving therapy decision-making of advanced tumors. Therefore, an overall improvement of prostate cancer management will need a comprehensive effort to devise new tools for patient stratification and prediction of therapy response. A future clinical use of miRs in the era of individualized oncology may satisfy the requirement for a patient-tailored therapeutic approach, based on personalized therapeutic choices guided by patient' molecular profiles. Biomarker signature for patient stratification and therapy decision is also expected to influence modern therapeutic approaches to prostate cancer treatment such as neo- or adjuvant systemic therapy, early chemotherapy, bisphosphonates, and targeted therapies. In fact, new targeted treatments such as Denosumab, Abiraterone, Sipuleucel-T, androgen receptor-, MET receptor-, and angiokinase-inhibitors will highly benefit from molecular biomarkers that support the decision-making process.

To date, an increasing number of published studies are examining the role of microRNAs as direct targets for prostate cancer therapy (Table 4). However, the majority of studies are based on prostate cancer cell line analysis which have no valuable clinical relevance.

### 4.1. Radiation Therapy

Radiation therapy (RT) is one of the treatment options for localized, high risk prostate cancer tumors which cannot be treated with radical prostatectomy. However, the risk of tumor regrowth following RT remains high for a number of cancer patients, despite modern radiation oncology techniques allowing specific delivery of high radiation doses directly to the tumor volume [105, 106]. Thus, radiation resistance remains an open issue to be solved. The role of microRNAs in RT is not yet fully understood and the few studies analyzing the effects of X-rays administration in changing miR expression profile come from basic research. In 2008, Josson and colleagues [107] observed a considerable downregulation of 6 miRs after irradiation of androgen-dependent LnCaP and androgen-independent LnCaP C4-2B cell lines. miR-521 was found to be downregulated to a greater extent in both cell lines and its forced expression increased LnCaP sensitivity to radiation-induced damages. The observed phenotype was demonstrated to be a consequence of Cockayne syndrome A (CSA), a DNA repair protein, and manganese superoxide dismutase (MnSOD), an anti-apoptotic and antioxidant enzyme, regulation. All these results suggest that miR-521 could be a new potential tool in enhancing the efficacy of radiation treatment in PCa.

LnCaP cells were used as* in vitro* model for measuring radiation effects also by Li et al. [108] who demonstrated that overexpression of miR-106b is sufficient to override the cell-cycle arrest induced by irradiation, through downregulation of its validated target p21 [109]. Interestingly, this microRNA was previously found upregulated in PCa specimens [32] compared to normal control. Altogether, these results suggest a potential therapeutic role of miR-106b suppression. Another microRNA involved in radiation therapy resistance is miR-95, which was found to be upregulated in radiation resistant PC3 cell line compared to parental control [110]. A further analysis correlated resistance acquisition with suppression of the sphingosine-1-phosphate phosphatase 1 (SGPP1) which was demonstrated to be directly regulated by miR-95.

Despite this recent advance, little is known about the regulatory effects of miRs on radiation resistance acquisition and the molecular mechanisms involved. Consequently, a comprehensive analysis on the role of microRNAs in treatment responsiveness, especially in the clinical setting, is still required to improve patient prognosis.

### 4.2. Hormone Therapy

Androgen deprivation therapy is the standard treatment for patients who experience recurrence after surgical resection of the prostate but, within two years after the initial treatment, a significant group of these patients develop incurable forms. Several microRNAs have been associated with castration-resistant properties of the cells through AR regulation. Overexpression of miR-21, which is positively modulated by androgen receptor, is able to increase proliferation abilities of androgen-dependent LnCaP and LAPC-4 cell lines, overcoming cell cycle arrest induced by testosterone deprivation and anti-AR treatment. Moreover, the effect of miR-21 in evasion of castration-mediated growth arrest was also confirmed* in vivo*, giving further evidence of its role in PCa [85]. These results are in agreement with translation studies which indicate miR-21 as one of the microRNAs upregulated to major extent in prostate cancer progression and suggest future implications for personalized target therapies.

The opposite scenario has been described for the tumor-suppressor miR-331-3p [111]. This microRNA is able to reduce v-erb-b2 avian erythroblastic leukemia viral oncogene homolog 2 (ERBB2) expression, a known oncogene whose expression increases in advanced prostate cancer [112], and to impair AR signaling pathway. Furthermore, miR-331-3p increases the bicalutamide-induced inhibition of PSA expression.

Although the significant results obtained for miR-21 and miR-331-3p, changing in their expression was not confirmed by Ottman and colleagues [113]. They analyzed different LnCaP-derived cell lines with different sensitiveness to androgen withdrawal and CDX treatment. Comparing microRNA expression profiles, they found 21 upregulated and 22 downregulated miRs in both androgen-deprivation and CDX administration conditions. Interestingly, deregulation of several microRNAs was also confirmed in a study conducted on patient samples derived from a neoadjuvant trial consisting of 8 men treated with goserelin, 9 men treated with CDX (*n* = 9), and 10 men untreated, prior to prostatectomy [114]. Analysis of resected tumor tissues revealed an upregulation of miR-9 and miR-17 and a downregulation of miR-218 in agreement with Ottman's study. Furthermore, the authors identified a panel of miRs upregulated (miR-141, -375, -210, etc.) and downregulated (miR-204, -100, -125b, etc.) after hormone treatment, whose expression trend correlates with poor prognosis.

Although we are moving toward translational medicine, most studies still rely on prostate cancer cell lines as surrogates for therapy response, which is not favorable. As the attempts to unravel the molecular mechanisms responsible for castration-resistant state are producing intriguingly results, it is evident that shifting this knowledge into the clinical setting will provide a great benefit for patient outcome.

### 4.3. Chemotherapy

Chemotherapy is offered to suitable patients with CRPC who have failed other treatment options. However, 40–50% of patients with CRPC do not respond substantially to chemotherapy, with the median duration of response being 6–9 months [115].

EMT is an event frequently involved in chemotherapy resistance of cancer cells. Puhr et al. [116] reported that docetaxel-resistant prostate cancer cells underwent an epithelial-to-mesenchymal transition during the selection process, leading to diminished E-cadherin levels and upregulation of mesenchymal markers. This phenotype was accompanied by a significant downregulation of miR-200c and miR-205 which, once reexpressed, were able to rescue E-cadherin and increase apoptotic rate of resistant cells. Different studies demonstrated the correlation of miR-205 with enhanced cisplatin cytotoxicity, through negative modulation of autophagic pathway, and with docetaxel-resistance acquisition [116, 117]. Docetaxel-resistant cell lines were also analyzed by Lin et al. in order to identify candidate circulating microRNA biomarkers able to predict chemotherapy responsiveness [118]. After a first screening in naïve and resistant PC3 and DU145 cell lines, the authors selected a panel of 46 deregulated microRNAs which were further analyzed for their expression in plasma and serum samples derived from 97 CRPC patients who were stratified in responder and nonresponder groups. Six microRNAs (miR-200c, miR-200b, miR-146a, miR-222, miR-301b, and miR-20a) were significantly associated with therapy responsiveness on the basis of their pretreatment levels and posttreatment expression changing. Furthermore, 12 microRNAs (miR-200b, -200c, -200a, -429, -21, -590-5p, -375, -132, -20a, -20b, -25, and -222) were correlated with patient overall survival.

In PCa, miR-34a and miR-148 were associated with paclitaxel resistance. Restored expression of miR-34a is able to reduce proliferative capabilities of PC3 taxol-resistant cells through modulation of sirtuin 1 (SIRT1) and antiapoptotic BCL2 [119]. Interestingly, a similar effect was observed by Rokhlin et al. [120], whereby simultaneous overexpression of miR-34a and miR-34c resulted in increased p53-mediated apoptosis in response to doxorubicin treatment of LNCaP cells. The role of miR-148a in the response of hormone-refractory prostate cancer cells to chemotherapy was investigated by Fujita and colleagues [121]. Expression levels of miR-148a were found to be lower in both PC3 and DU145 hormone-refractory prostate cancer cells, compared to normal human prostate epithelial cells and LNCaP hormone-sensitive prostate cancer cells. Furthermore, forced expression of miR-148 in PC3 cells inhibited cell growth, cell migration, and cell invasion and increased sensitivity to paclitaxel through modulation of MSK1.

The theoretical concept of EMT is strictly correlated with the new evidence demonstrating the presence within the tumor mass of a stem-like subset of cells which are able to self-renew and drive cancer development and progression [122]. Moreover, increasing evidence indicates that these cells, which are called cancer stem cells (CSCs), are responsible for drug resistance, tumor recurrence, and metastasis formation [123, 124]. CSCs hypothesis suggests several possible explanations for the mainly unsolved questions of treating patients with cancer, such as local recurrence after treatment of solid tumor by radiation or chemotherapy and development of metastases that can appear many years after curative surgical treatment of primary tumor. The first who analyzed the role of microRNAs in stem-like prostate cancer cells were Liu and colleagues in 2011 (Table 5) [125]. They demonstrated that forced expression of miR-34a reduces purified CD44^+^ stem-like prostate cancer cells and that it is sufficient to inhibit clonogenic expansion, tumor regeneration, and metastasis. In contrast, downregulation of miR-34a in CD44^−^ cell lines promoted tumor development and invasive properties. Furthermore, the authors identified and validated CD44 itself as a direct and functional target of this microRNA. CD44 protein is also a direct target of the tumor-suppressor miR-708. As a consequence of this regulation, ectopic expression of miR-708 is able to inhibit the tumor-initiating capacity of prostate cancer cells* in vitro* and to reduce tumor progression in prostate cancer xenografts [126]. A further analysis revealed that low miR-708 expression was associated significantly with poor survival outcome, tumor progression, and recurrence in patients with prostate cancer. Finally, miR-320, -143, -145, and let-7 were also associated with suppression of stem-like properties of the cells [125, 127–129].

Taken together, these results establish a strong rationale for developing microRNA-based therapy for targeting prostate CSCs in order to eradicate the basal core of tumors and restore patient responsiveness to current pharmacological treatment.

## 5. Conclusion

In the last decade, the advent of PSA screening improved PCa detection but its low predictivity caused overdiagnosis and overtreatment with consequent increase in patient morbidity [5]. In addition, analysis of PSA blood levels does not represent* per se* an unequivocal method to assess the presence of prostate adenocarcinoma since it can be influenced by nonneoplastic alterations. Thus, patients necessarily undergo multiple local biopsies which up to now represent the standard approach for diagnosis. However, it is possible that also after anatomical and pathological evaluation of tissue biopsies, diagnosis still remains uncertain with consequent psychological stress for the patients. In this context, a lot of effort has been dedicated trying to improve our capabilities to manage cancer diseases and the exponential development of innovative technologies creating the bases for translating results from basic research to a real patients' clinical benefit. Thus, the role of microRNAs in prostate cancer provides a solid rationale for their further evaluation in clinical practice. In fact, microRNAs' ability to regulate almost all cellular processes makes them an attractive tool for disease management, including cancer. Furthermore, their high stability in patient-derived tissues and body fluids provides the possibility to perform noninvasive screening which are able to evaluate patients' natural history of the disease with high accuracy, due to the fact that microRNAs are representative of all tumor molecular changing during the time. The increasing sensitiveness and reliability of new technologies assure the analysis starting from low amount of material giving the opportunity to create new accurate and noninvasive tests. In particular for prostate cancer, the identification of microRNA signatures, correlating specifically with tumor properties, represents a new source of specific biomarkers and the future advantage for both diagnosis and prognosis is clearly evident. The increasing amount of studies analyzing miR expression profiles is a consequence of this assumption with the specific aim to identify innovative biomarkers able to distinguish nonneoplastic alteration of the prostate from localized tumors and indolent from aggressive tumors, in order to decrease the incidence of overdiagnosis/overtreatment and to guide therapeutic decision, respectively. In the era of personalized therapy, it is imperative to find new biomarkers able to mirror cancer aberrant molecular setting and to guarantee monitoring cancer progression during early and late stages. Thus, the possibility to create a personal molecular profiling of the tumors may determine the creation of an individual therapeutic protocol optimizing therapy benefit and reducing secondary effects of nonnecessary treatment. Finally, advancement in delivery systems (liposomes and nanoparticles among others) and molecule stability (LNA-modified anti-miR and microRNA-mimics) have paved the way for the use of microRNAs as effective drugs for integrated therapy [130, 131] and several clinical trials testing the efficacy of microRNAs are ongoing. This field may offer a great opportunity in terms of application in tumor disease, including PCa. Overall, improving prostate cancer management will provide both individual and social benefits due to the massive social impact of this malignancy, allowing at the same time a significant rationalization of financial resources dedicated by the national health system.

## Figures and Tables

**Table 1:** MicroRNAs associated with PCa diagnosis.

References	Sample type	miRs deregulated	miRs selected as candidate biomarkers	
Taylor et al. (2010) [24]	113 PCa tissues 28 normal tissues	Large screening study	Taylor et al. 10.1016/j.ccr.2010.05.026	

Volinia et al. (2006) [31]	56 PCa tissues 7 normal tissues	39 upregulated 6 downregulated	Volinia et al. 10.1073/pnas.0510565103;	

Ambs et al. (2008) [32]	60 microdissected tumor tissues 16 normal tissues	21 upregulated 21 downregulated	miR-32, miR-26a, miR-181a, miR-93, miR-196a, miR25, miR-92 and let-7i	↑

Porkka et al. (2007) [38]	5 hormone-naïve PCa tissues 4 HRPC tissues 4 BPH tissues	14 upregulated 37 downregulated	PCa: miR-16, miR-99 and let-7 family	↓
			HRPC: miR-205, miR-100 and miR-30	↓

Ozen et al. (2008) [43]	16 PCa tissues and 10 normal tissues	9 upregulated 76 downregulated	Let-7, miR-30, miR-16	↓

Martens-Uzunova et al. (2012) [44]	102 PCa tissues and normal adjacent tissues	54 deregulated	miR-205	↓

Larne et al. (2013) [45]	49 PCa tissues 25 normal tissues	7 deregulated	miR-96-5p, miR-183-5p	↑
			miR-145-5, miR-221-5p	↓
			(combined in miQ score)	

Moltzahn et al. (2011) [46]	Serum samples from PCa (*n* = 36) and HD (*n* = 12)	6 upregulated 4 downregulated	miR-20b, miR-874, miR-1274a, miR-1207-5p, miR-93, miR-106a	↑
			miR-223, miR-26b, miR-30c, miR-24	↓

Bryant et al. (2012) [48]	Plasma samples from PCa (*n* = 78) and HD (*n* = 28) Urine samples from PCa (*n* = 118) and HD (*n* = 17)	12 deregulated	miR-107, miR-574-3p	↑

Srivastava et al. (2013) [49]	40 PCa tissues and 40 normal adjacent tissues. Urine samples from PCa (*n* = 36) and HD (*n* = 12)	2 downregulated	miR-205, miR-214	↓

Haj-Ahmad et al. (2014) [50]	Urine samples from PCa (*n* = 8) BPH (*n* = 12) patients and HD (*n* = 10)	17 deregulated (only 7 selected for further analysis)	miR-1825 (only in PCa)	↑
			miR-484 (in PCa and BPH)	↓

Schaefer et al. (2010) [54]	76 PCa and adjacent normal tissues	5 upregulated 10 downregulated	miR-96, miR-182, miR-182∗ miR-183 and miR-375	↑
			miR-16, miR-31, miR-125b, miR-145, miR-149, miR-181b, miR-184, miR-205, miR-221, miR-222	↓

PCa: prostate cancer; HRPC: hormone refractory prostate cancer; BPH: benign prostatic hyperplasia; HD: healthy donors, FFPE: formalin-fixed, paraffin-embedded.

**Table 2:** MicroRNAs associated with PCa prognosis.

References	Sample type	Clinical parameters	miRs deregulated	miRs selected as candidate biomarkers	
Taylor et al. (2010) [24]	113 PCa tissues 28 normal tissues	Large screening study		Taylor et al. 10.1016/j.ccr.2010.05.026	

Martens-Uzunova et al. (2012) [44]	102 PCa tissues and normal adjacent tissues	High risk biochemical recurrence	12 upregulated 13 downregulated	miR-19a, miR-130a, miR-20a/106/93	**↑**
				miR-27, miR-143, miR-221/222	**↓**

Tong et al. (2009) [53]	40 FFPE prostatectomy Specimens (20 without early BCR 20 with early BCR)	Biochemical recurrence	2 upregulated 4 downregulated	miR-135, miR-194 (40% of case)	**↑**
				miR-145, miR-221, miR-222	**↓**

Schaefer et al. (2010) [54]	76 PCa and adjacent normal tissues	Biochemical recurrence	5 upregulated 10 downregulated	miR-96	**↑**

Hulf et al. (2013) [55]	149 PCa and 30 matched normal tissues	Biochemical recurrence	1 downregulated	miR-205	**↓**

Schubert et al. (2013) [62]	BCR tissues and disease-free tissues	Biochemical recurrence	2 downregulated	let-7b and let-7c	**↓**

Leite et al. (2011) [66]	21 frozen BCR tissues 28 frozen disease-free tissues	Biochemical recurrence	4 upregulated	miR-100	**↑**

Karatas et al. (2014) [72]	82 PCa tissues (41 BCR and 41 disease-free)	Biochemical recurrence	3 downregulated	miR-1, miR-133b	**↓**

Selth et al. (2013) [102]	Serum samples from PCa patients (BCR = 8) disease-free = 8)	Biochemical recurrence	3 upregulated	miR-194 miR-146-3p	**↑**

Shen et al. (2012) [87]	Plasma samples from PCa (*n* = 82)	Castration resistance	2 upregulated	miR-21, miR-145	**↑**

Jalava et al. (2012) [90]	28 primary PCa tissues 14 CRPC tissues 12 BPH tissues	Castration resistance	4 upregulated 3 downregulated	miR-32, miR-148a, miR-590-5p, miR-21	**↑**
				miR-99a, miR-99b, miR-221	**↓**

Peng et al. (2011) [94]	6 primary PCa tissues 7 bone metastatic tissues	Metastasis	5 downregulated	miR-508-5p, miR-143, miR-145, miR-33a, miR-100	**↓**

Saini et al. (2011) [95]	36 PCa tissues 8 metastatic tissues 8 normal tissues	Metastasis	1 downregulated	miR-203	**↓**

Mitchell et al. (2008) [19]	Serum samples from metastatic PCa (*n* = 25) and age-matched HD (*n* = 25)	Metastasis	6 deregulated	miR-141	**↑**

Brase et al. (2011) [100]	Serum samples from localized PCa (*n* = 14) and metastatic PCa (*n* = 7)	Metastasis	5 upregulated	miR-141, miR-375	**↑**

Bryant et al. (2012) [48]	Serum samples from PCa (*n* = 72) and metastatic PCa (*n* = 47) Plasma samples from PCa (*n* = 55) and metastatic PCa (*n* = 24)	Metastasis	2 upregulated	miR-141 and miR-375	**↑**

Nguyen et al. (2013) [103]	Serum samples from localized PCa (*n* = 58) and metastatic CRPC (*n* = 26)	Castration resistance	3 upregulated 1 downregulated	miR-141, miR-375, miR-378∗	**↑**
				miR-409-3p	**↓**

Zhang et al. (2011) [88]	Serum samples from localized PCa (*n* = 20), ADPC (*n* = 20), CRPC DTX treated (*n* = 10) and BPH (*n* = 6)	Castration resistance	1 upregulated	miR-21	**↑**

FFPE: formalin-fixed, paraffin-embedded, BCR: biochemical recurrence, Pca: prostate cancer, CRPC: castration resistant prostate cancer, BPH: benign prostatic hyperplasia, HD: healthy donors.

**Table 3:** MicroRNAs regulating AR expression through direct targeting.

References	miRs involved in AR regulation
Hagman et al. (2013) [57]	miR-205
Qu et al. (2013) [132]	miR-185
Lin et al. (2013) [133]	miR-31
Sikand et al. (2011) [134]	miR-488∗
Östling et al. (2011) [135]	miR-135b, miR-105b, miR-297,
	miR-299-3p, miR-34a, miR-34c, miR-371-3p, miR-421, miR-499a, miR-499b, miR-634, miR-654-5p, miR-9

**Table 4:** MicroRNAs associated with PCa therapy.

References	Sample type	Therapy	miRs deregulated	miRs selected as candidates	
Josson et al. (2008) [107]	LnCaP and LnCaP C4-2B	Radiation therapy	6 downregulated	miR-521	↓

Huang et al. (2013) [110]	PC3 radiation resistant cells	Radiation therapy	1 upregulated	miR-95	↑

Ribas et al. (2009) [85]	LNCaP and LAPC-4	Hormone therapy	Overexpressed	miR-21	↑

Ottman et al. (2014) [113]	LnCap CDX sensitive cells LnCap CDX non-sensitive cells	Androgen deprivation-therapy and casodex	21 upregulated 22 downregulated	http://www.molecularcancer.com /content/13/1/1	

Lehmusvaara et al. (2013) [114]	28 tumor tissues (*n* = 8 goserelin-treated patients *n* = 9 bicalutamide-treated patients *n* = 11 no treated-patients)	Endocrine treatment	10 deregulated	miR-9 and miR-17	↑
				miR-218	↓

Zhang et al. (2011) [88]	Serum samples from localized PCa (*n* = 20), ADPC (*n* = 20), CRPC DTX treated (*n* = 10) and BPH (*n* = 6)	Docetaxel	1 upregulated	miR-21 (in CRPC docetaxel resistant)	↑

Puhr et al. (2012) [116]	PC3 docetaxel-resistant cells	Docetaxel	2 downregulated	miR-200c, miR-205	↓

Lin et al. (2014) [118]	Serum and plasma samples from CRPC PCa (*n* = 97) before and after therapy	Docetaxel	46 deregulated	miR-200c, miR-200b, miR-146a,	
				miR-222, miR-301b, miR-20a	

Kojima et al. (2010) [119]	PC3 paclitaxel-resistant cells	Paclitaxel	1 downregulated	miR-34a	↓

Fujita et al. (2010) [121]	PC3 and DU145	Paclitaxel	1 downregulated	miR-148	↓

PCa: prostate cancer; ADPC: androgen dependent prostate cancer; CRPC: castration resistant prostate cancer; BPH: benign prostatic hyperplasia; DTX: docetaxel; CDX: casodex.

**Table 5:** MicroRNAs associated with stemness properties acquisition.

References	miRs associated with stemness properties
Liu et al. (2012) [125]	miR-34a
Saini et al. (2012) [126]	miR-708
Huang et al. (2012) [127]	miR-143 and miR-145
Hsieh et al. (2013) [129]	miR-320
